# The dynamic interplay between viruses and host non-coding RNA species

**DOI:** 10.1093/femsre/fuag003

**Published:** 2026-02-09

**Authors:** Lorraine Matandirotya, Lauren Burke, Ella Dimascio, Sarah Menezes, Katie Allott, Adrian Whitehouse

**Affiliations:** School of Molecular and Cellular Biology, Faculty of Biological Sciences, University of Leeds, Leeds LS2 9JT, United Kingdom; School of Molecular and Cellular Biology, Faculty of Biological Sciences, University of Leeds, Leeds LS2 9JT, United Kingdom; School of Molecular and Cellular Biology, Faculty of Biological Sciences, University of Leeds, Leeds LS2 9JT, United Kingdom; School of Molecular and Cellular Biology, Faculty of Biological Sciences, University of Leeds, Leeds LS2 9JT, United Kingdom; School of Molecular and Cellular Biology, Faculty of Biological Sciences, University of Leeds, Leeds LS2 9JT, United Kingdom; School of Molecular and Cellular Biology, Faculty of Biological Sciences, University of Leeds, Leeds LS2 9JT, United Kingdom; University of Leeds, Astbury Centre for Structural Molecular Biology, Leeds LS2 9JT, United Kingdom

**Keywords:** non-coding RNA, viruses, host, gene regulation, disease

## Abstract

Previously understudied for their lack of protein-coding capacity and assumed functional irrelevance, non-coding RNAs (ncRNAs) have emerged as crucial regulators of biological pathways. Technological advancements, including optimized RNA sequencing methods have begun unearthing the extent of ncRNA contributions to cellular processes; however, many ncRNAs remain partially characterized. Nevertheless, ncRNAs have cemented their role as crucial regulators of gene expression, reinforced by ncRNA dysregulation being implicated in the development and progression of a wide range of human diseases. Viruses have evolved intricate mechanisms to override host immune strategies and propagate viral replication. Many of these involve manipulating host ncRNA networks or encoding viral ncRNA species to fine-tune the cellular milieu into one most permissive for viral biology. Yet, due to their regulatory potential, ncRNAs are also integral to cellular immune strategies and defence mechanisms, such that ncRNAs remain one of the main tools hosts use to subdue viral infections. Herein we describe the complex and dynamic interplay between viruses and host non-coding regulatory RNA species. We characterize the various classes of ncRNAs in comprehensive detail and explore their respective contributions to viral biology. We then discuss the therapeutic potential of ncRNAs, and their putative roles as specific biomarkers.

## Teaser

Review of the different classes of non-coding RNA, and the complex interplay between viruses and host non-coding regulatory RNA species. Ceated in BioRender. Whitehouse, A. (2026) https://BioRender.com/22vb2aa.

## Introduction

Pervasive transcription refers to the phenomenon whereby the majority of the human genome (∼80%) is transcribed, yet only a meagre 1.5% of these transcripts are translated. Though they do not encode proteins, several of these residual transcripts, termed non-coding RNAs (ncRNAs), are functional RNA molecules with regulatory roles in multiple cellular processes (Birney et al. 2007). Initially, ncRNAs were regarded as ‘genomic junk’ upon their discovery. They have since been redefined as canonical factors in gene regulation, acting both independently and in dynamic complexes to influence global gene expression. The multi-level influence of ncRNAs on chromatin remodelling, transcriptional, and post-transcriptional regulation results in ncRNAs impacting an array of biological and pathological functions (Kaikkonen et al. 2011, Liu et al. 2021, Statello et al. 2021).

ncRNAs fall into two main groups based on their length; small ncRNAs, which are shorter than 200 nucleotides, and long ncRNAs (lncRNAs), which are 200 nucleotides or longer (Bhat et al. 2020). Small ncRNAs are broadly classified into two main groups; functional and regulatory ncRNAs. Technological advances have allowed for more elaborate characterization of small ncRNAs, and these are further subdivided based on their heterogeneity in terms of function, source, and conformation. Amongst the small functional ncRNAs are ribosomal RNAs (rRNAs), transfer RNAs (tRNAs), small nuclear RNAs (snRNAs), and small nucleolar RNAs (snoRNAs). Small regulatory RNAs include microRNAs (miRNAs), small interfering RNAs (siRNAs), piwi-interacting RNAs (piRNAs), and tRNA fragments (tRFs).

The ubiquitous and diverse nature of ncRNAs affords them an extensive target profile, which, in turn, solidifies their physiological relevance. The biological importance of ncRNAs in regulatory networks has become increasingly evident, with numerous studies implicating them in genomic integrity maintenance, chromatin remodelling, and protein synthesis (Cox and Sullivan 2014, Gong and Maquat 2011, Tycowski et al. 2015). As such, it is unsurprising that their dysregulation has been noted in various diseases such as neurodegeneration, cardiovascular disorders, cancer, and viral infections.

Although the full extent of ncRNA contribution to pathophysiology is still being elucidated, particularly from infectious diseases, irrefutable evidence has highlighted the role of ncRNAs in an array of viral infections. To enhance their own survival, viruses can hijack and disrupt cellular ncRNA networks or introduce their own virus-encoded ncRNA to the infected host. Virus-encoded ncRNA also provide an effective immune-evasion strategy during host entry, as their lack of protein-coding capacity prevents the generation of surface peptides that would otherwise be recognized by the major histocompatibility complex on T-cells and activate an adaptive immune response (Tagawa et al. 2021, Wieczorek et al. 2017). Both methods can confer a significant advantage for viruses, which are often limited by their small genome size.

The versatility and regulatory prowess of ncRNAs has them often caught in the middle of a complex host-viral interplay, where both the host and infecting virus recruit ncRNA species to drive their own survival. As such, ncRNA species often function in antagonistic pro- and antiviral capacities (Bermudez-Santana and Gallego-Gomez 2024). In this review, we characterize various ncRNA species implicated in viral biology and categorically discuss their roles in different viral infections, focusing primarily on ncRNAs with experimentally validated roles in viral replication and host defence. We conclude by discussing the potential use of ncRNA as therapeutic strategies and prognostic biomarkers in antiviral medicine.

## microRNAs

miRNAs are small, highly conserved non-coding RNA molecules that are between 21 and 22 nucleotides in length and play key roles in post-transcriptional gene regulation. As such, aberrant miRNA expression is associated with a wide variety of pathophysiological conditions (Adams et al. 2014, Colpaert and Calore 2019, Karnati et al. 2015). miRNAs function by recognizing and binding to complementary sequences in the 3’ UTR of target mRNAs to induce their degradation or translational repression (Ha and Kim 2014). A small subset of miRNAs have unique target binding sites within 5’UTRs and coding regions; however, these alternative functional routes still lead to transcript degradation or the translational repression of target mRNAs (Broughton et al. 2016, Lytle et al. 2007).

## miRNA biogenesis

**Figure 1:**
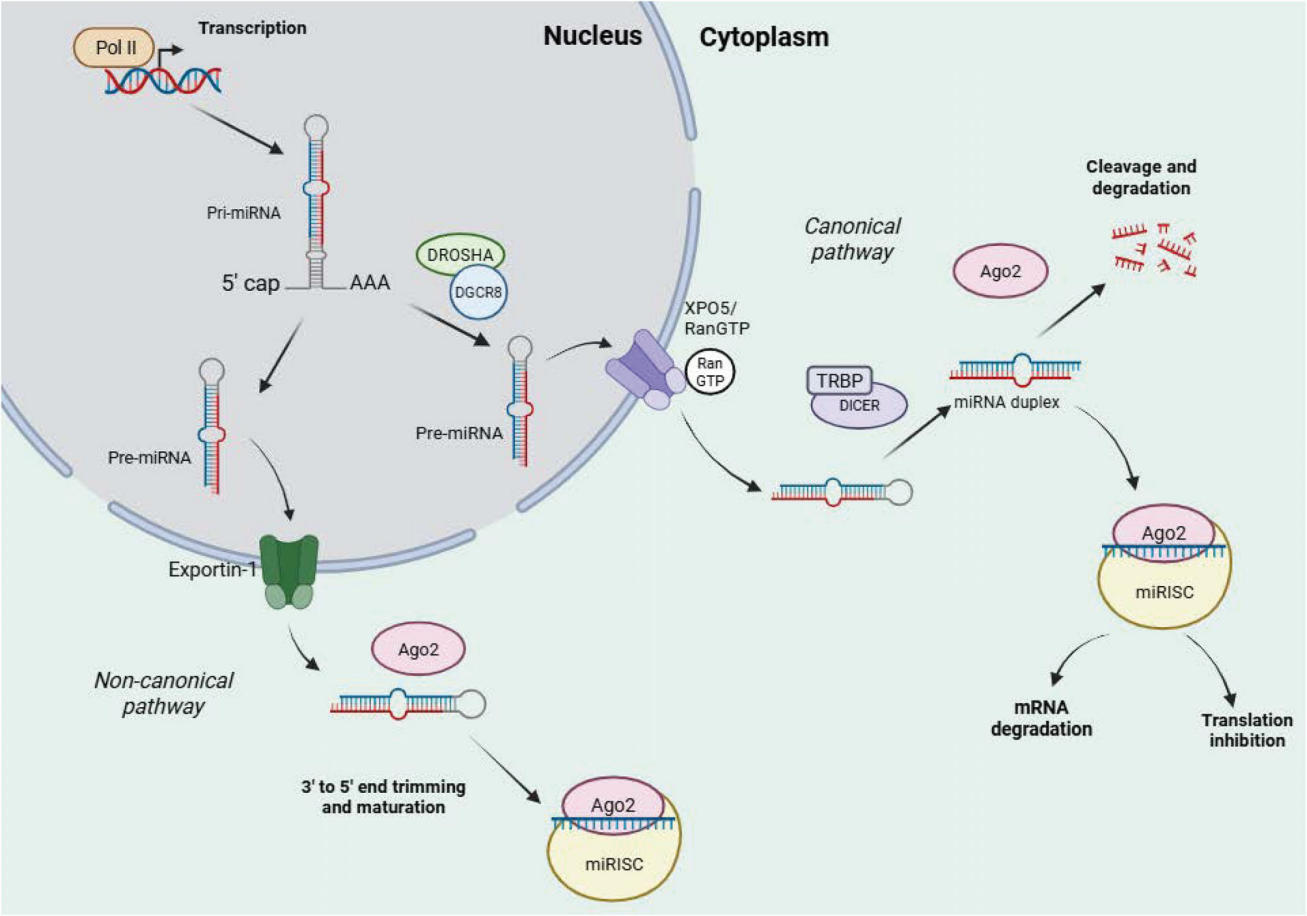
miRNA biogenesis pathways. miRNA biogenesis occurs via one of two pathways: canonical or non-canonical biogenesis. Both pathways begin with transcription of pri-miRNA by RNA polymerase II. In the canonical pathway, pri-miRNAs are processed to pre-miRNA by the Drosha-DGCR8 microprocessor complex. Pre-miRNAs are then exported to the cytoplasm for Dicer-mediated cleavage into two strands. The guide strand is loaded into the RISC complex to form the miRISC complex. This interacts with Ago to form a mature miRNA. The remaining strand is cleaved and degraded. In the non-canonical pathway, biogenesis occurs in a Drosha/DGCR-8- or Dicer-independent manner. Pre-miRNAs generated without Drosha/DGCR8 resemble Dicer substrates, allowing for their export to the cytoplasm for further processing. Created in BioRender. Whitehouse, A. (2026) https://BioRender.com/o3b3kx6.

miRNA biogenesis begins in the nucleus with the transcription of hairpin primary miRNAs (pri-miRNAs) by RNA polymerase II (Ha and Kim 2014). Maturation then proceeds via one of two pathways—the canonical or non-canonical pathway (Fig. 1).

### The canonical pathway

pri-miRNAs are processed into precursor miRNA molecules (pre-miRNA) by Drosha ribonuclease III (Drosha) and DiGeorge syndrome chromosomal region 8 (DGCR8), which form the Drosha-DGCR8 microprocessor complex. Drosha cleaves the pri-miRNA to produce a 3’ overhang, whilst DGCR8 plays a role in recognizing and binding pri-miRNAs, particularly the hairpin structures and flanking single-stranded RNA segments. It also helps to stabilize Drosha and ensures accurate cleavage of the pri-miRNA, releasing the pre-miRNA precursor. Once generated, pre-miRNAs are exported into the cytoplasm by the exportin-5 (XPO5)/Ran GTP complex, where they are cleaved into two strands by the endonuclease Dicer (Denli et al. 2004, Okada et al. 2009). One strand, termed the guide strand, is loaded into the RNA-induced silencing complex (RISC) to form a miRNA-induced silencing complex (miRISC), and interacts with Argonaute (Ago) family proteins in an ATP-dependent manner to form a mature miRNA (Yoda et al. 2010). Meanwhile, the remaining passenger strand is cleaved by Ago2 and degraded by cellular machinery (Ha and Kim 2014).

### The non-canonical pathway

Non-canonical miRNA biogenesis occurs in a Drosha/DGCR8- or Dicer-independent manner. The pre-miRNAs generated during Drosha/DGCR8-independent biogenesis resemble Dicer substrates, allowing for their direct export into the cytoplasm via exportin-1 (O’Brien et al. 2018). During Dicer-independent biogenesis, Droshamediated processing generates pre-miRNAs that are too small to be Dicer substrates; therefore, the full-length pre-mRNA molecule is loaded onto Ago2 in the cytoplasm, where it undergoes 3’-5’ end trimming and maturation (Cheloufi et al. 2010).

## Target RNA-directed miRNA degradation

Under normal conditions, miRNAs target mRNA for post-transcriptional repression through degradation (Orang et al. 2014). However, certain miRNA targets contain ‘triggers’ that induce the proteolytic cleavage of Ago proteins, which in turn causes the rapid degradation of their associated miRNAs. This results in a unique miRNA repression mechanism called targeted RNA-directed miRNA degradation (Ameres et al. 2010).

Herpesvirus saimiri (HVS) was the first virus shown to target miRNAs in this manner. HVS encodes seven small nuclear RNAs, called HSURs, which contain miRNA ‘triggers’. HSUR-1 targets the T-cell repressor miR-27 for degradation (Guo et al. 2014). In so doing, HSUR-1 prolongs T-cell activation, which in turn contributes to HVS infection and oncogenesis (Guo et al. 2014, Wightman et al. 2018). Similarly, HSUR2 binds to miR-142–3p and miR-16, both of which are involved in the G1-S cycle transition. This promotes host cell entry into the S phase to create a more conducive environment for HVS replication (Cazalla et al. 2010). This mechanism has also been observed in murine cytomegalovirus (MCMV) and human cytomegalovirus (HCMV) infection (Buhagiar and Kleaveland 2024).

## miRNAs and viruses

### Herpesviruses

The vast majority of virus-encoded miRNAs are harboured within the herpesviridae family (Table 1), an unsurprising consequence of the biphasic lifecycle of these viruses. The ability of herpesviruses to cause long-term persistent infections in their hosts necessitates long-term immune-evasion strategies, and virus-encoded miRNAs are thought to be key players in this process (Carden et al. 2024).

**Table 1:** Summary of virus-associated miRNA.

Virus	miRNA	Mechanism/function	Reference
HVS	HSUR-1 (V)	Targets miR-27 for degradation through TDMD. This leads to prolonged T-cell activation, contributing to HVS infection and oncogenesis	PMID: 24 725 595 PMID: 30 333 855
	HSUR-2 (V)	Degrades miR-142–3p and miR-16 through TDMD. Promotes host cell entry to S phase, driving HVS replication	PMID: 31 039 329
HSV-1	HSV-miR-H6 (V)	Downregulates transcription factor ICP4. Prevents HSV-1 gene expression to maintain latency	PMID: 22 550 533 PMID: 18 596 690
	HSV-1-miR-H2-3p (V)	Downregulates transcription factor ICP0. Prevents HSV-1 gene expression to maintain latency	PMID: 18596690
KSHV	miR-K12-9 (V) miR-K12-7-5p (V)	Downregulates RTA. Prevents latent to lytic switch to maintain KSHV latency	PMID: 20 006 845 PMID: 21 283 761
	miR-K12-11 (miR-155 homologue) (V)	miR-155 homologue, dysregulates TBF-β signalling and promotes viral infection by modulating the innate immune response	PMID: 22 013 049 PMID: 21 221 132
	miR-132 (C)	Downregulates p300 expression levels and regulates innate antiviral immunity, with effects on KSHV gene expression	PMID: 30 951 791
HCMV	miR-17 (C, down) miR-20a (C, down)	HCMV induces downregulation of miR-17 and miR-20a and enhances viral replication by dysregulating cell cycle progression	PMID: 23 768 492
EBV	miR-BART2 (V)	Targets DNA polymerase BALF5 to inhibit DNA replication and maintain viral latency	PMID: 18 073 197
	miR-BART20-5p (V)	Suppresses BAD-induced caspase-3-dependent apoptosis to prevent aberrant activation of lytic replication	PMID: 26 581 978
	miR-BART22 (V)	Plays a role in nasopharyngeal carcinoma and is correlated with poor patient prognosis and metastasis	PMID: 35 907 914
HBV	miR-122 (C, down)	HBV induces downregulation of miR-122, causing increased HBV infection, hepatitis and HCC progression	PMID: 29 973 597
HCV	miR-122 (C, up)	Aids IRES formation for viral protein translation, masks HCV genomic RNA and prevents degradation	PMID: 29 973 597
SARS-CoV-2	CoV2-miR-O8 (V)	Predicted to target IFN-1 signalling pathway	PMID: 38 226 175
	CoV2-miR-O7a (V)	Mechanism of action remains to be elucidated, but expression correlates with increased viral load and suppressed cellular miRNA activity	PMID: 34 903 581
	miR-150–5p (C, up)	Upregulated in asymptomatic patients, downregulated in intensive care patients. Possibly involved in immune system regulation	PMID: 3 513 828 PMID: 30 984 166
EBOV	miR-MAY-251 (V) miR-MAK-40 (V)	Targets genes involved in cellular regulation, particularly in the PANTHER pathway.Targeting of genes involved in haemorrhagic EBOV phenotype, while roles in regulation of virus replication and host immune defence modulation	PMID: 35 563 619
	NC_060925.1:203674252..203674274 (C)	Significantly upregulated by EBOV to promote Axon guidance, cell adhesion, virus entry, host immune responses, virus infection and pathogenesis	PMID: 38 674 337
HIV	miR-H3 (V)	Targets HIV 5’ LTR and activates viral transcription by upregulating promoter activity	PMID: 24 620 741
	miR-H1 (V)	Targets and downregulates cellular miR-149 to prevent miR-149 from degrading the VpR gene, which is important for the virus lifecycle	PMID: 21 786 074 PMID: 24 158 819
	miR-198 (C, up)	HIV induces downregulation of miR-198, which is thought to have antiviral activity. This prevents suppression of HIV replication and gene expression	PMID: 19 148 268

***Key: (C = cellular, V = viral, up = upregulated, down = downregulated)***

Viral miRNAs were first discovered in 2004 when Pfeffer and colleagues uncovered the regulatory capacity of small Epstein-Barr virus (EBV)-derived miRNA species (Pfeffer et al. 2004). At least 44 mature EBV-encoded miRNAs have since been identified, the majority of which originate from the BART cluster (Barth et al. 2011).

EBV miRNAs facilitate viral persistence, immune evasion, and anti-apoptotic pathways during latent replication. Not surprisingly, miRNAs are highly expressed during latency to support these crucial functions (Albanese et al. 2016, Barth et al. 2008, 2011, Pfeffer et al. 2004). miR-BART2 promotes latent infection by inhibiting the lytic DNA polymerase, BALF5, and obstructing EBV lytic gene replication in this manner (Barth et al. 2008). Similarly, miR-BART20-5p prevents caspase-3-mediated lytic induction and enhances cell survival via the targeted inhibition of the pro-apoptotic protein, BAD (Kim et al. 2015). EBV-encoded miRNAs also orchestrate immune evasion strategies, some of which involve targeting the pro-inflammatory cytokine IL-12, or inhibiting TAP2; a crucial component of the MHC class I pathway. Both mechanisms culminate in poor host recognition of CD8+ EBV-infected T-cells, which promotes pathogen persistence (Albanese et al. 2016).

Kaposi’s sarcoma-associated herpesvirus (KSHV) employs similar mechanisms to sustain latency. KSHV encodes miR-K12-9 and miR-K12-7-5p (Bellare and Ganem 2009), both of which downregulate the replication and transcription activator (RTA), the latent-lytic switch protein that is necessary and sufficient to induce KSHV lytic replication (Lin et al. 2011). Herpes simplex virus 1 (HSV-1) encodes HSV-1-miR-H2-3p and HSV-1-miR-H6, both of which maintain viral latency by repressing the transcription factors ICP0 and ICP4, respectively. ICP0 and ICP4 both promote lytic replication, so their combined repression effectively maintains HSV-1 latency (Everett 2000, Halford et al. 2001, Umbach et al. 2008).

Herpesviruses infamously dysregulate host miRNAs for their own benefit. The cellular miR-155 is a central player in multiple regulatory pathways, modulating humoral and adaptive immune responses and promoting lymphocyte proliferation. The functional versatility of miR-155 has attracted considerable attention from the gammaherpesvirus subfamily, with both EBV and KSHV co-opting means to exploit its activity (Liang et al. 2011, Liu et al. 2012, Wood et al. 2018). EBV upregulates miR-155 expression to modulate innate immune responses and facilitate viral replication (Wood et al. 2018). Meanwhile, KSHV adopts a different approach, encoding its own viral homologue of miR-155 to dysregulate TGF-β signalling and promote viral infection (Liang et al. 2011, Liu et al. 2012). This homologue has an identical seed sequence to cellular miR-155, enabling target specificity and efficient regulation of cognate cellular target genes (Skalsky et al. 2007).

To subvert host detection, KSHV also upregulates cellular miR-132 to downregulate cognate p300 expression levels (Hussein et al. 2019, Lagos et al. 2010). Interestingly, cellular miRNAs such as miR-3164 and miR-3130–5p are dysregulated as swiftly as 15 minutes post KSHV infection. This rapid dysregulation of miRNA networks highlights how effectively viruses are able to modulate host gene expression (Hussein and Akula 2017).

HCMV drives viral replication by dysregulating miRNA expression, albeit through miRNA attenuation. In contrast, HCMV specifically drives the degradation of miR-17 and miR-20a to accelerate viral production. Both miR-17 and miR-20a regulate genes involved in cell cycle progression and apoptotic pathways. In consequence, their targeted degradation during HCMV infection alters normal cell cycle progression and modifies the cellular environment into one more favourable for viral replication (Lee et al. 2013).

### HBV and HCV

Hepatitis B and Hepatitis C viruses (HBV and HCV) are hepatotropic viruses that cause acute and chronic cirrhosis and are primary drivers of hepatocellular carcinoma (HCC) (Li and Lo 2015). miR-122 is a liver-specific tumour suppressor with multiple complementary viral mRNA targets (Chang et al. 2004, Lagos-Quintana et al. 2002, Wen and Friedman 2012, Xu et al. 2020).

During HBV infection, miR-122 exhibits antiviral activity by binding to conserved regions within HBV pre-genomic RNA (pgRNA) and mRNA. This negatively impacts the viral polymerase and targets the 3’UTR of the core protein, thus downregulating pgRNA stability and viral protein expression and, in consequence, HBV replication (Chen et al. 2011, Qiu et al. 2010). Moreover, miR-122 modulates host pathways and acts as a tumour suppressor by downregulating Cyclin G1, which in turn increases p53 activity. This reduces HBV transcription, and contributes to the overall inhibition of HBV replication (Wang et al. 2012). As such, HBV restricts miR-122 to aid in its own infection and replication. Studies have confirmed that HBV infection is correlated with miR-122 downregulation, with lower miRNA levels contributing to hepatitis and HCC progression (Li et al. 2013, Mahmoudian-Sani et al. 2019, Song et al. 2015).

Interestingly, miR-122 plays a paradoxical role in HCV infection, where it is crucial for successful viral replication and propagation (Schult et al. 2018). miR-122 acts as a chaperone, binding to two conserved sites located at the 5’ end of the HCV genome to assist in the formation of internal ribosome entry sites (IRES) that are essential for viral protein translation (Schult et al. 2018). Moreover, miR-122 ‘capping’ of the 5’ end masks HCV genomic RNA and prevents degradation by host 5’ exonucleases, aiding immune evasion, but also enhancing viral protein production (Schult et al. 2018, Shimakami et al. 2012).

### Arboviruses

RNA interference (RNAi) is the predominant defence strategy against RNA viruses in insects and plants (Kakumani et al. 2013). The dengue virus (DENV)-encoded protein NS4B circumvents host RNAi systems by interfering with the processing of Dicer, which in turn shuts down the miRNA machinery and impedes host immune responses to infection (Kakumani et al. 2013). Relatedly, DENV infection also abrogates the mRNA levels of RNAi-associated factors such as Drosha, Ago1, and Ago2 (Kakumani et al. 2013).

Subgenomic flaviviral RNAs (sfRNAs) are ncRNA species generated from the incomplete degradation of viral genomic RNA by the cellular exonuclease, XRN1 (Chapman et al. 2014). DENV sfRNA species accumulate during infection and function in a similar way to NS4B, disrupting Ago2 and Dicer function (Moon et al. 2015). Conserved sfRNA activity is observed in West Nile virus (WNV) infection. WNV encodes a sfRNA, which disrupts RISC complexation and assembly, causing a decrease in antiviral miRNA levels, enhancing arboviral replication in insect cells (Schnettler et al. 2012).

### SARS-CoV-2

A significant number of miRNAs are differentially expressed following SARS-CoV-2 infection, and the unique miRNA signature encompassing miR-150–5p, miR-375, and miR-122–5p can serve as a determinant of patient prognosis in some cases (Akula et al. 2022, Fernandez-Pato et al. 2022). Interestingly, miR-150–5p, a miRNA involved in immune regulation and lymphogenesis (Cron et al. 2019) is upregulated in asymptomatic COVID patients (Fernández-Pato et al. 2022), whereas decreased miR-150–5p levels correlate with an increased intensive care unit stay, suggesting miR-150–5p upregulation may facilitate immune system regulation to prevent exaggerated immune responses and severe illness (Kim et al. 2021, Morán et al. 2015).

SARS-CoV-2 also expresses its own miRNAs, such as CoV2-miR-O8. Although its exact function remains to be elucidated, CoV2-miR-O8 shows clear miRNA structure and is processed by miRNA biogenesis proteins. CoV2-miR-O8 is predicted to target genes associated with the IFN-1 signalling pathway; however, further validation is still required (Tucker et al. 2024). An additional SARS-CoV-2 miRNA, CoV2-miR-O7a, positively correlates with increased viral load, and suppresses cellular miRNA activity (Pawlica et al. 2021).

### Ebola virus

Ebola virus (EBOV) encodes EBOV-miR-MAY-251 and miR-MAK-403 (from the Mayinga and Makona variants, respectively). Both miRNAs target genes involved in the regulation of cellular processes, particularly in the PANTHER signalling pathway (Diallo et al. 2022). A recent study showed that EBOV also dysregulates the novel miRNA, NC_060925.1:203674252..203674274, which targets genes associated with Axon guidance, MAPK signalling, and FoxO signalling, to facilitate virus entry and immune evasion (Mensah-Bonsu et al. 2024).

### Retrovirus

It is apparent that viral miRNAs are predominantly encoded by DNA viruses, likely due to the resource-rich nuclear localization of most DNA viruses granting them access to vital manufacturing and processing machinery. The evolutionary disadvantages potentially associated with encoding miRNA species in RNA viruses may also account for the lower prevalence, as these miRNA would pose an inherent threat to viral RNA stability due to the risk of self-recognition. Nevertheless, at least one retrovirus has demonstrated miRNA coding capacity; bovine leukaemia virus (BLV) (Kincaid et al. 2012). In contrast, there is controversy over whether the lentivirus HIV also encodes miRNAs. Original reports suggested the presence of several HIV-encoded miRNAs with pro-viral roles in promoting viral transcription and directly targeting antiviral miRNAs (Kaul et al. 2009, Pilakka-Kanthikeel et al. 2011, Zhang et al. 2014, Zhao and Bukrinsky 2014). However, parallel studies have failed to detect these miRNAs (Lin and Cullen 2007).

A summary of all the miRNAs highlighted above, selected for their computationally predicted or experimentally validated roles in viral replication and host defence, are included in Table 1.

### piRNAs

Piwi-interacting RNA (piRNA) represent an animal-specific class of regulatory small ncRNA that bind to Ago proteins to exert their silencing capacity in a manner similar to miRNA. However, piRNAs harbour distinct characteristics. Unlike miRNA that are processed from dsRNA precursors in a Dicer-dependent manner to produce 20–22 nt mature miRNA molecules, piRNAs are generated from single-stranded precursor transcripts in a Dicer-independent manner to form slightly longer (24–31 nt) molecules. Furthermore, piRNAs form effector complexes with a germline-specific class of Ago proteins known as the p-element induced wimpy testis (piwi) subfamily (Aravin et al. 2006, Girard et al. 2006, Haase 2022, Ozata et al. 2019).

### piRNA biogenesis

piRNA biogenesis is a unique and intricate process that occurs in three main steps: primary generation of precursors, secondary processing, and tertiary maturation (Malone et al. 2009, Ozata et al. 2019).

#### Primary biogenesis

**Figure 2:**
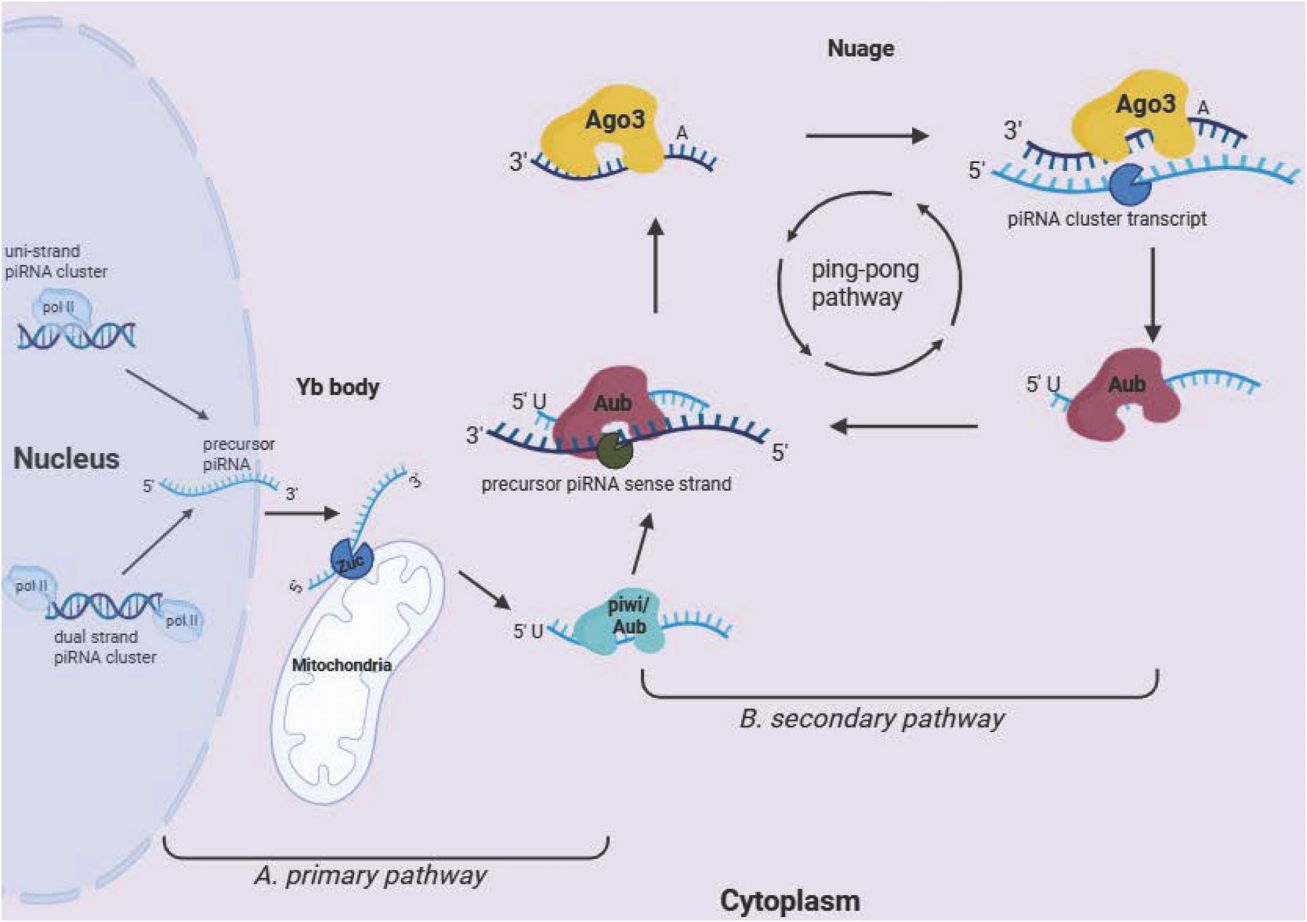
piRNA biogenesis pathways. (A) Primary piRNA pathway: in the nucleus, precursor piRNAs are transcribed from single-stranded and dual-stranded piRNA clusters by RNA polymerase II. piRNA precursors are then transported to Yb bodies near the mitochondrial surface for processing by the enzyme Zuc. The piRNA intermediates are loaded onto piwi proteins to create piRNA-piwi complexes or loaded onto Aub and fed into the ping-pong pathway. (B) Ping-pong pathway: piRNA-Aub complexes bind and cleave complementary precursor transcripts. The cleavage products are loaded onto Ago3, and these complexes in turn recognize and cleave their complementary substrates, generating more piRNA species. Created in BioRender. Whitehouse, A. (2026) https://BioRender.com/vvyj171.

The majority of piRNAs are expressed from discrete genomic loci termed piRNA clusters, subclassified as uni- or dual-stranded clusters based on their use of either one or both DNA strands as the transcriptional template (Mohn et al. 2014). Once transcribed, precursor piRNAs are transported to specialized compartments near the mitochondrial surface known as Yb bodies for processing. Here, the endonuclease Zucchini (Zuc) cleaves and gives them their characteristic 5’ uridine monophosphate (1 U) signature (Ipsaro et al. 2012, Malone et al. 2009, Rayford et al. 2021). Next, primary piRNAs are loaded onto piwi proteins to execute their downstream transcriptional silencing, or they are loaded onto the Aubergine (Aub) protein for secondary biogenesis via the ping-pong cycle (Fig. 2) (Czech and Hannon 2016).

#### The ping-pong cycle

In compartments known as nuage bodies, Aub binds antisense strand piRNAs, and the Aub-piRNA complex recognizes and cleaves complementary sense transposons at the 10th nucleotide, creating sense-strand piRNAs, which bind Ago3. The sequence complementarity between Ago-bound and Aub-bound piRNAs enables them to continuously cleave their respective precursor substrates and generate new piRNAs (Fig. 2) (Czech and Hannon 2016, Gunawardane et al. 2007). In this manner, the secondary ping-pong loop enables the swift amplification of the piRNA pool (Czech and Hannon 2016). Ago3-bound piRNA typically contain an adenine residue at the 10th nucleotide, and as such, 10A bias is a characteristic signature of piRNA biogenesis via the ping pong cycle (Fig. 2) (Brennecke et al. 2007, Nemeth et al. 2024).

#### Tertiary maturation

Tertiary maturation is mediated by the exonuclease Nibbler, which facilitates piRNA 3’ end processing. A subsequent maturation step adds a terminal 2’-O-methyl residue that contributes to piRNA stability (Lewis et al. 2018).

### piRNA functions

The most widely reported function of piRNA is the germline-specific silencing of transposable elements. Somatic functions are less well characterized, but of increasing interest is their emerging role as immunogenic drivers in arboviral vectors (Iwasaki et al. 2015, Kolliopoulou et al. 2019).

Transposable elements present a major threat to the integrity of the genome due to their ability to replicate and reinsert at different genomic loci, often altering exonic sequences and intronic splicing patterns (Iwasaki et al. 2015). piRNAs are the major line of defence against transposable elements. The mode of transposon repression is species-specific but typically occurs through transcriptional silencing and direct mRNA cleavage. Transcriptional silencing occurs when piRNA-guided piwi proteins bind to nascent transposon transcripts to promote transcript cleavage (Brennecke et al. 2007, Lewis et al. 2018, Ozata et al. 2019). In flies, piwi also promotes H3K9 methylation, a repressive chromatin mark which reprograms transcription from the generation of functional, spliced transposons to the generation of precursor piRNA, and redirects these to the piRNA biogenesis workflow. In so doing, transposons are ingeniously reprogrammed into piRNA-generating loci (Blair 2019, Thomas et al. 2014).

### piRNA and viruses

An exciting recent discovery is the role of piRNAs in antiviral defence mechanisms (Table 2). This function is most inferable in the context of single-stranded viral genomes, as the structural similarity between ssRNA and piRNA substrates makes them excellent piRNA targets. In some instances, the host resourcefully uses the viral genome to prime itself against attack by repurposing the virus and feeding it into the piRNA pathway, such that it recognizes and targets itself for degradation (Kolliopoulou et al. 2019). Extensive studies have corroborated the antiviral role of piRNAs in mosquitoes, with similar accounts of a somatic accumulation of virus-specific piRNA-like following alphavirus, bunyavirus, and flavivirus infection (Dietrich et al. 2017, Göertz et al. 2019, Kolliopoulou et al. 2019, Morazzani et al. 2012).

**Table 2:** Summary of virus-associated piRNA.

Virus	piRNA/piRNA protein	Mechanism/function	Reference
RSV	piR-38587 (C, up)	Targets adaptin proteins for degradation, potentially dysregulating cytoskeletal/Golgi organization.	PMID: 36 158 569
HSV	piRNA-hsa-28 382 (C, up)	Predicted to target genes involved in cell growth and proliferation. This causes a decrease in HSV viral titres and inhibits viral propagation.	PMID: 36 894 069
SARS-CoV-2	PIWIL2 protein (C, down)	Downregulates the levels of exosomal piRNAs and reduces antiviral efficacy of exosomes. Promotes viral replication.	PMID: 34 862 272
HPV	FR018916, FR140858, FR197104, FR237180, FR298757 (C, up)	Increased expression of piRNA signature is associated with poor patient prognosis.	PMID: 26 852 287

***Key: (C* *=* *cellular, V* *=* *viral, up* *=* *upregulated, down* *=* *downregulated)***

A possible antiviral role of piRNA in Drosophila has also been proposed; however, whether or not it exists is still polarizing, as conflicting studies have been documented in the literature. Zambon et al. (2006) reported that loss of function mutations in key piwi pathway genes (piwi, aubergine, armitage, and vasa) suppressed the immune response to Drosophila X virus (DXV) in adult flies, leading to increased fly death. Similarly, Chotkowski et al. (2008) reported that mutation of piwi led to increased virion production in WNV-infected flies. Conversely, upon conducting loss of function mutations in zuc, piwi, aubergine, and Ago3, Petit et al. (2016) reported no significant changes in fly susceptibility to Drosophila C Virus (DCV) or DXV, and concluded that piRNA failed to play an antiviral role in Drosophila, attributing the conflicting findings previously reported in literature to misinterpretation of genetic variance between wild type and mutants used in the studies.

### RSV and HSV

To investigate a potential role of piRNA in respiratory syncytial virus (RSV) infection, Corsello and colleagues conducted a piRNA microarray analysis in RSV-infected human small airway epithelial cells and discovered that piRNA are dysregulated in a time-dependent manner upon RSV infection (Corsello et al. 2022). Subsequent *in silico* gene ontology studies conducted on the upregulated piRNAs identified an enrichment in pathways involving cytoskeletal/Golgi organization, providing a potential link between piRNA and viral defence, as cytoskeletal reorganization has long been implicated in RSV virion assembly and budding (Li et al. 2024, Linfield et al. 2021, Shahriari et al. 2016). A parallel study determining piRNA function in the context of HSV infection also reported the differential expression of piRNA, with gene ontology and KEGG analyses implicating piRNAs in signalling pathways and antiviral immune responses. Furthermore, overexpression of their top target piRNA-hsa-28 382 caused a prominent reduction in viral titres in HSV-infected human fibroblasts (Wang et al. 2023).

### SARS-CoV-2

Using *in vitro* models of SARS-CoV-2 infection, murine neuronal stem cells were shown to release piRNA-enriched exosomes/microvesicles to trigger an innate immune antiviral response. Knockout of the PIWIL2 protein reversed this phenotype, suggesting that the piRNA-piwi system is integral to the functionality of these antiviral exosomes (Ikhlas et al. 2022).

### HPV

Human papillomavirus (HPV) is causally linked to intraepithelial neoplasia and represents the most significant risk factor in the development of cervical cancer. HPV is also a major oncogenic driver of head and neck squamous cell carcinoma (HNSCC) (Li et al. 2024). A key study conducted by Firmino et al. (2016) identified distinct piRNA expression patterns between HPV-positive HNSCC and non-malignant tissues, with the expression of 41 piRNAs being uniquely dependent on HPV infection status. Furthermore, they identified a unique piRNA signature, as the combined expression of five specific piRNAs was associated with poor patient survival. Their findings were paramount, as the unique piRNA signature highlighted the potential use of piRNA as biomarkers (Firmino et al. 2016).

### LncRNA

**Figure 3:**
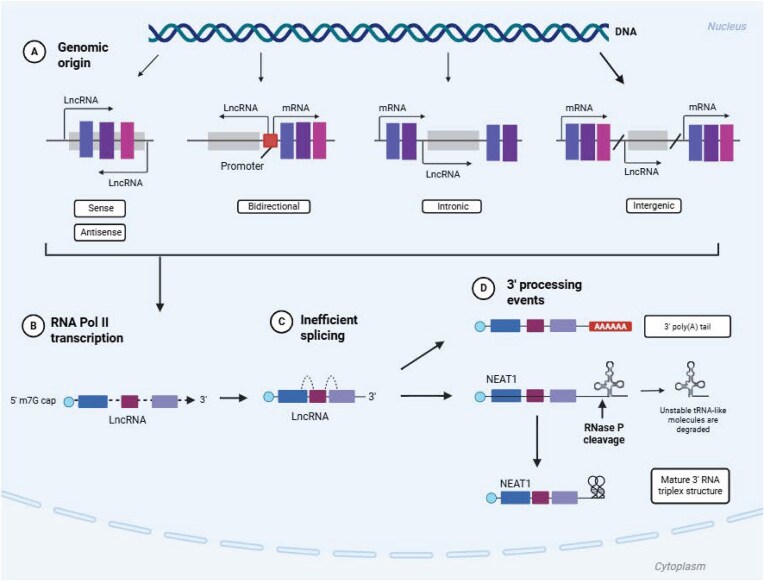
Biogenesis of lncRNAs. (A) LncRNA transcription occurs via multiple pathways from sense and antisense strands of protein-coding genes. Bidirectional transcription from the promoter of a protein-coding gene in the opposite direction. Intronic transcription occurs in intronic regions of protein-coding genes. Intergenic—the lncRNA is transcribed from regions between two protein-coding genes. (B) Most lncRNAs are transcribed by RNA polymerase II and are 5’ capped. (C) Alternative splicing occurs in most lncRNAs; however, this is less efficient compared to mRNA splicing. (D) Most lncRNAs are 3’ polyadenylated; however, some can undergo unique 3’ processing events, for example, NEAT1 3’ ends are processed by ribonuclease P to produce tRNA-like RNA products. Created in BioRender. Whitehouse, A. (2026) https://BioRender.com/c7ngp4s.

LncRNAs are highly abundant in the human genome, with GENCODE estimating the existence of over 20 000 encoded lncRNAs (Frankish et al. 2023). Advances in transcriptomic technologies, such as capture long-read RNA sequencing, continue to expand this catalogue (Statello et al. 2021). Like mRNA, most lncRNAs are transcribed from RNA polymerase II, contain multiple exons, are alternatively spliced, 5’ capped, and 3’ polyadenylated. However, some lncRNAs are processed from intronic or intergenic regions of both sense and antisense strands of protein-coding genes and can undergo unique 3’ end processing (Fig. 3) (Mattick et al. 2023, Quinn and Chang 2016). LncRNA expression exhibits high levels of cellular tropism, yet they display lower sequence conservation and expression levels than protein-coding genes. Despite this, lncRNAs have evolved into multi-faceted molecules, with expansive roles in chromatin and subnuclear structural organization, epigenetic gene regulation, and cell signalling. More recently, lncRNAs have emerged as important regulators of viral infection and the antiviral immune response.

## Structure-function relationship of lncRNA

LncRNA modes of action appear more heavily reliant on secondary and tertiary structure than primary sequence conservation (Graf and Kretz 2020). The ‘RNA modular code’ hypothesis suggests that lncRNAs have complex structural sub-domains which fold into distinct nodes, enabling them to act as guides, scaffolds, and molecular decoys for their targets (Guttman and Rinn 2012). For example, the lncRNA GAS5 has three distinct structural elements, each with independent functions in regulating cell survival. The 3’ stem loop structure interacts with steroid receptors (SRs) to block DNA-dependent steroid signalling, the core module mediates effects on mammalian target of rapamycin (mTOR) inhibition on cell growth, and the 5’ module affects basal survival independent of SRs (Frank et al. 2020). Overall, it is clear lncRNA architecture is critical to lncRNA function.

### Functions of lncRNA

Functionally, lncRNA can act as molecular scaffolds to bring multi-complex proteins into proximity, bind to promoter regions of protein-coding genes to modulate transcriptional apparatus, stabilize DNA through the formation of R loops, and act as sponges for microRNAs (Shi et al. 2022).

#### Chromatin regulation

Several lncRNAs localize to the nucleus and are highly chromatin associated (Douka et al. 2021). A well-annotated lncRNA, telomerase RNA template component (TERC), is essential for chromatin maintenance due to its contribution to the catalytic activity of telomerase reverse transcriptase (Mefford et al. 2020). The lncRNA X-inactivation specific transcript (XIST) plays a critical role during mammalian embryonic cell development, where it silences X chromosomal genes for dosage compensation (Loda et al. 2022).

#### Formation of subnuclear structures

LncRNAs are indispensable during the formation of subnuclear bodies such as paraspeckles. Paraspeckles are membrane-less organelles formed by liquid-liquid phase separation (LLPS) and have significant roles in gene regulation (Lin et al. 2018). The lncRNA, NEAT1, is an essential scaffold in nuclear paraspeckle formation, allowing stable binding of the key paraspeckle proteins, NONO and SFPQ (Naganuma and Hirose 2013).

### lncRNAs and viruses

The expansive regulatory roles of lncRNAs render them essential mediators for both infecting viruses and cellular hosts alike. Unsurprisingly, numerous lncRNAs are differentially expressed following viral infection, either to boost host defence mechanisms, or drive viral propagation (Meng et al. 2017, Peng et al. 2010, Zhang et al. 2016) (Table 3). Viruses have also evolved to encode their own lncRNAs to enhance pathogenicity (Li et al. 2022). Viral lncRNAs disrupt host gene expression by dysregulating the stability of host mRNA. Prominently, flavivirus sfRNAs are not only resistant to the exonuclease, XNR1, but have been shown to inhibit XNR1 activity, leading to an accumulation of unstable host mRNAs and defective immune activation (Moon et al. 2012).

**Table 3:** lncRNAs dysregulated during viral infection.

Virus	lncRNA	Function/mechanism	Reference
EMCV, HCV, HBV	lncRNA #32 (C, down)	Stabilized by hnRNPU and binds ATF2 to promote transcription of IGSs.	PMID: 27 582 466
LCMV	Morrbid (C, up)	Modulates the response of CD8+ T cells by promoting the expression of the proapoptotic gene, *Bcl2l11*, and downregulating of the PI3K–AKT pathway.	PMID: 31 138 702
VSV, VACV, HSV-1	lncRNA-ACOD1 (C, up)	Enhances the activity of metabolic enzyme glutamic-oxaloacetic transaminase (GOT2), increasing viral replication.	PMID: 29 074 580
HCV	GAS5 (C, up)	Binds with HCV NS3 protein, suppressing viral replication by acting as a protein decoy.	PMID: 26 945 984
HCV, SFV, IAV	EGOT (C, up)	Activated by PKR and RIG-I signalling, suppresses expression of IGSs resulting in enhanced viral replication.	PMID: 27 283 940
IAV	NRAV (C, down)	Suppresses the IGSs IFIT2, IFIT3, IFITM3, and MxA by regulating histone modification of ISGs.	PMID: 25 525 793
	lnc-IGS20 (C, up)	Lnc-IGS20 competitively binds to miR-326; lnc-ISG20 sequesters the miRNA away to allow for the accumulation of ISG20 protein levels, which elicits a more effective immune response.	PMID: 29 899 085
	IPAN (C, up)	Stabilizes the viral polymerase protein PB1 by forming an IPAN/PB1 complex, which prevents PB1 degradation and ensures efficient viral RNA synthesis.	PMID: 31 189 112
	TSPOAP1-AS1 (C, up)	Induced by NF-κB signalling, negatively regulates type I IFN signalling and suppresses expression of the ISGs Ifit1, Ifitm3, Oasl, and ISG20, enhances viral replication.	PMID: 30 968 963
IAV, VSV	VIN (C, up)	Nuclear-enriched host factor essential for IAV replication.	PMID: 24 440 876
HIV-1	NRON (C, down)	NRON represses proviral transcription factor NFAT. Viral protein Nef downregulates NRON early in infection, restores NFAT activity, and enhances viral replication.	PMID: 25 728 138
KSHV, HIV-1	NEAT1 (C, up)	KSHV induces NEAT1-containing virally modified paraspeckles (V-mPS). These V-mPS are essential hubs for processing viral transcripts. HIV-1 infection downregulates NEAT1. Reduction in NEAT1 led to export of unspliced, instability element (INS)-containing HIV-1 mRNAs, which enhanced virus production.	PMID: 39 592 606 PMID: 23 362 321
KSHV	PAN RNA (V)	Facilitates lytic reactivation through interactions with histone demethylases, UTX, and JMJD3, leading to removal of repressive H3K27me3 marks from the ORF50 promoter. Acts as a molecular scaffold for the viral proteins, ORF50 and ORF57, bringing RNA pol II into proximity to viral promoters. Downregulates the expression of host immune factors such as gamma interferon, interleukin-18, alpha interferon 16, and RNase L by inhibiting cellular transcription factors. Interacts polycomb repression complex 2 (PRC2) to hinder the production of inflammatory cytokines.	PMID: 22 589 717 PMID: 32 143 650 PMID: 21 957 289 PMID: 23 468 496
EBV	BHLF1 (V)	Forms a stable RNA-DNA hybrid at the OriLyt site during lytic replication, needed for loading viral DNA-binding protein, BALF2, and initiation of DNA replication.	PMID: 21 191 028
HSV-1	LAT (V)	Maintains viral latency through suppression of reactivation by blocking the function of early genes and having an anti-apoptotic effect on host cells.	PMID: 27 055 281
Flaviviruses	sfRNAs (V)	Dysregulate the stability of host mRNAs, inhibits exonuclease XNR1 activity, leading to an accumulation of unstable host mRNAs.	PMID: 23 006 624

***Key: (C* *=* *cellular, V* *=* *viral, up* *=* *upregulated, down* *=* *downregulated)***

Many lncRNA transcripts are regulated by the interferon response and are involved in the expression of interferon-stimulated genes (Carpenter et al. 2013, Josset et al. 2014, Kambara et al. 2014, Zhou et al. 2019). For example, lncRNA #32 contributes to antiviral immune responses by interacting with the transcription factor ATF2 to enhance the transcription of ISGs (Nishitsuji et al. 2016). Silencing of lncRNA #32 leads to diminished ISG expression, which is associated with increased titres of viruses such as EMCV, HCV, and HBV. More recently, lncRNAs have been implicated in adaptive immunity, particularly in the differentiation and function of virus-specific CD8⁺ T-cells (Hudson et al. 2019). Notably, the lncRNA Morrbid modulates CD8⁺ T-cell responses to LCMV infection by promoting expression of the pro-apoptotic gene, *Bcl2l11*, and repressing PI3K–AKT signalling, thereby fine-tuning T-cell expansion and effector activity (Kotzin et al. 2019).

LncRNAs are frequently co-opted by viruses to promote a seamless progression through the viral life cycle. The interferon-independent lncRNA-ACOD1 is upregulated by multiple viruses such as vaccinia virus (VACV), VSV, and HSV-1 for its ability to manipulate host metabolic networks. LncRNA-ACOD1 interacts with and stimulates the metabolic enzyme glutamic-oxaloacetic transaminase (GOT2), and the subsequent increase in GOT2 metabolites facilitates viral replication and infection (Chen and Chang 2017, Wang et al. 2017). The potential for lncRNA translation during viral infection is an emerging area, with evidence supporting the formation of virus-induced specialized ribosomes (Harrington et al. 2025, Murphy et al. 2023).

### IAV

Host lncRNAs typically impede IAV replication by upregulating interferon-stimulated genes (ISGs). Conversely, the host lncRNA Negative Regulator of Antiviral Response (NRAV) displays unique pro-viral potential. Overexpression of NRAV was shown to promote IAV replication by suppressing the initial transcription of ISGs IFIT2, IFIT3, IFITM3, and MxA, likely through modulating histone modifications at their promoters. In consequence, NRAV is promptly downregulated during IAV replication as a host antiviral response to limit viral replication (Ouyang et al. 2014).

ISG20 is a 3′-5′ RNA exonuclease with broad antiviral activity against several RNA viruses, including IAV (Deymier et al. 2022). LncRNA-interferon-stimulated gene 20 (lnc-ISG20), is a cellular lncRNA that also disrupts IAV replication by enhancing ISG20 protein expression. Under physiological conditions, miR-326 represses ISG20 activity. Due to the sequence homology between ISG-20 and lnc-ISG20, the lncRNA can competitively bind to miR-326. In so doing, lnc-ISG20 sequesters the miRNA away to allow for the accumulation of ISG20 levels, which elicits a more effective immune response (Chai et al. 2018).

In contrast, IAV-induced dysregulation of cellular lncRNAs has been implicated in productive viral replication. The host lncRNAs VIN and TSPOAP1-AS1 are both enriched in the nucleus during IAV infection and are essential for viral replication, acting through IFN-independent mechanisms and active suppression of IFN signalling, respectively (Wang et al. 2022, Winterling et al. 2014). Moreover, the interferon-independent lncRNA, IPAN, is specifically induced by IAV infection and is a crucial host factor for IAV replication. IPAN stabilizes the viral polymerase protein PB1 by forming an IPAN/PB1 complex, which prevents PB1 degradation and ensures efficient viral RNA synthesis (Wang et al. 2019).

Compelling evidence suggests that lncRNAs can contain small open reading frames (smORFs), which encode micropeptides, some of which have distinct functional roles during viral infection. Novel data suggests that the micropeptide PESP, encoded by lncRNA PCBP1-AS1 is upregulated during IAV infection and contributes to viral replication. Mechanistically, PESP promotes autophagy; a pathway that is hijacked and subverted by IAV to promote virion assembly (Chi et al. 2024).

### EBV

The EBV-encoded lncRNA, BHLF1, forms a stable RNA-DNA hybrid at the origin of replication (OriLyt) site during lytic replication. This is essential for loading of the viral DNA-binding protein, BALF2, and initiation of DNA replication at the OriLyt (Rennekamp and Lieberman 2011).

Although they are not technically classified as lncRNAs for failure to meet the 200-nt threshold used during standard classification, EBER1 and EBER2 are highly abundant, 170-nt-long ncRNAs that accumulate in the nuclei of latently infected cells. EBER1 is suggested to promote cell proliferation by enhancing metabolic activity, possibly through regulation of mitochondrial activity (Ahmed et al. 2021). EBER2 supports EBV-driven B-cell transformation by enhancing the expression UCHL1, a de-ubiquitinase that drives expression of key cell-cycle modulators; Aurora kinases and cyclin B1 (Li et al. 2021). Furthermore, EBER2 regulates EBV gene expression by indirectly interacting with the host transcription factor PAX5 at viral genomic sites, modulating transcription of genes such as LMP2 (Lee et al. 2016). Together, EBER1 and EBER2 contribute to EBV oncogenesis by conferring resistance to interferon-α-induced apoptosis. This was originally linked to inhibition of PKR activation, although the exact mechanism in vivo has been debated (Elia et al. 2004, Nanbo 2002, Ruf et al. 2005). Recent evidence suggests both RNAs are trafficked out of the cell via exosomes, indicating a potential role in intercellular communication (Ahmed et al. 2018).

### KSHV

KSHV encodes the polyadenylated nuclear RNA (PAN), a multifunctional lncRNA that is abundantly expressed during lytic replication. PAN RNA regulates lytic induction, gene expression, and immune evasion. PAN RNA activates lytic replication through direct interaction with the viral genome and the histone demethylases UTX and JMJD3. This interaction leads to the removal of repressive H3K27me3 marks from the ORF50 promoter, which is necessary for its transactivation. This process is critical for the transition from viral latency to lytic replication (Rossetto and Pari 2012).

PAN RNA also acts as a molecular scaffold for the viral proteins ORF50 and ORF57. It facilitates the formation of genomic loops to bring cellular transcription machinery, such as RNA pol II, into proximity with distal viral promoters (Campbell and Izumiya 2020). This is necessary for efficient expression of the KSHV genome.

The versatility of PAN RNA is further exemplified by its ability to antagonize antiviral immune responses. PAN RNA downregulates the expression of host immune factors such as gamma interferon, interleukin-18, alpha interferon 16, and RNase L by inhibiting cellular transcription factors. Moreover, PAN RNA also interacts with the chromatin-modifying complex, polycomb repression complex 2 (PRC2) to hinder the production of inflammatory cytokines (Rossetto et al. 2013, Rossetto and Pari 2011).

KSHV also exploits cellular lncRNA function through drastically altering canonical NEAT1-containing paraspeckles during lytic replication to produce virally modified paraspeckles (V-mPS). These V-mPS function as hubs for processing viral transcripts and are essential for KSHV lytic replication (Harper et al. 2024).

### HCV

HCV exploits the cellular lncRNA EGOT to suppress IFN signalling and ISG production (Carnero et al. 2016). In contrast, GAS5 is upregulated during HCV infection and exerts antiviral effects by binding to the viral protein NS3, acting as a protein decoy to inhibit viral replication (Qian et al. 2016).

### HSV

The latency-associated transcript (LAT) represents one of the earliest described viral lncRNA. LAT is abundantly expressed in sensory neurones upon HSV-1 infection, as it is central to the latency-reactivation cycle (BenMohamed et al. 2015, Bloom et al. 1994, Carpenter et al. 2015, Peng et al. 2003). LAT neuronal expression correlates with attenuated levels of lytic genes, and early studies proposed that LAT maintained viral latency by facilitating heterochromatin deposition on lytic promoters to drive transcriptional silencing (Wang et al. 2005). However, this mechanism is contested in literature, as Kwiatkowski and colleagues observed that LAT promoter deletions produced contradictory effects on heterochromatinization (Kwiatkowski et al. 2009). Although the precise role of LAT in epigenetic regulation is yet to be fully elucidated, recent studies suggest LAT tethers viral episomes to the nuclear periphery through interactions with the host protein, TMEM43 (Grams et al. 2023).

Interestingly, LAT plays a paradoxical role in epithelial tissues, where the LAT locus supports lytic replication and serves as a determinant of HSV-1 virulence during lytic infection (Vanni et al. 2020). Small LAT-derived RNAs also demonstrate anti-apoptotic activity, independent of the canonical latency-reactivation function (Oh et al. 2024). Together, these findings highlight the multifaceted, and context-dependent roles of LAT in HSV-1 infection.

### HIV

NFAT is a transcription factor with important regulatory roles in HIV-1 replication but can be repressed by the cellular lncRNA NRON. HIV-1 counteracts this by producing an accessory protein, Nef, that downregulates NRON early in infection. This, in turn, restores NFAT activity and enhances viral replication (Imam et al. 2015).

As mentioned previously, the cellular lncRNA NEAT1 is a key element of nuclear paraspeckles. Studies show that NEAT1 is significantly dysregulated during viral infection. Zhang et al. (2013) showed that NEAT1 was consistently upregulated during HIV-1 infection. Subsequent knockdown of NEAT1 disrupted paraspeckle formation and led to increased nucleus-to-cytoplasm export of unspliced, instability element (INS)-containing HIV-1 mRNAs, which enhanced virus production (Zhang et al. 2013).

### circRNAs

Circular RNAs (circRNAs) are a novel type of ncRNA characterized by a covalently closed loop structure. Despite lacking both 5′ 7-methyl-guanosine cap and 3′ polyadenylation modifications, circRNAs are highly stable molecules with inherent resistance to exonuclease degradation (Kristensen et al. 2019). Initially described as ‘scrambled exons’, circRNAs were largely believed to be the result of splicing errors (Cocquerelle et al. 1993). Only upon the advent of next-generation sequencing methods, more than 20 years after their initial discovery, were circRNAs entertained as having functional capacity (Maass et al. 2017, Salzman et al. 2013, Wang et al. 2014). circRNAs have since been reported in every eukaryotic system (Wang et al. 2014) and their functional potential was acknowledged upon the discovery of their cell-specific expression (Maass et al. 2017, Salzman et al. 2013, Starke et al. 2015), and they are often differentially expressed relative to their linear counterparts (Salzman et al. 2013).

### circRNA biogenesis

**Figure 4:**
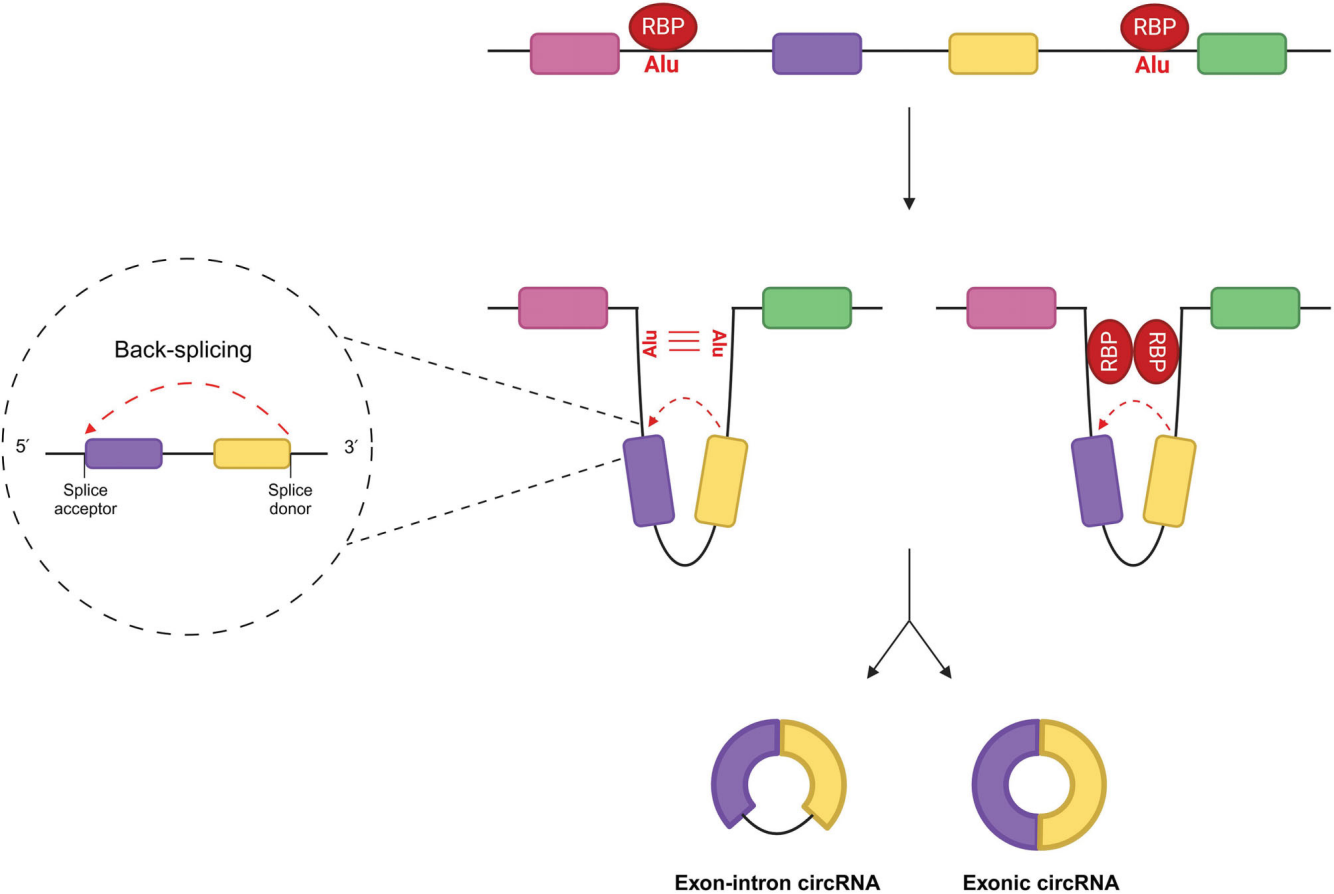
circRNA biogenesis pathways. circRNAs are formed via a unique back-splicing mechanism. Back-splicing involves the covalent joining of a downstream splice donor site to an upstream splice acceptor site, inducing circularization of the RNA. Back-splicing is favoured over canonical splicing when back-splice sites are brought into close proximity. This can occur due to base pairing of flanking repeat sequences (Alu) or following dimerization of bound RNA-binding proteins. Multiple exons and introns may be incorporated into the circularized RNA. Exonic circRNA is produced following additional canonical splicing events within the circularized RNA. Created in BioRender. Whitehouse, A. (2026) https://BioRender.com/z85l083.

circRNAs are formed via a unique splicing event known as back-splicing. Mechanistically, back-splicing involves the covalent joining of a downstream splice donor site to an upstream splice acceptor site, resulting in circularization of the RNA (Chen and Yang 2015) (Fig. 4). As both circRNAs and linear mRNA transcripts are produced from the same canonical splice sites within the same parental source gene, a competitive interplay exists between the two mechanisms (Ashwal-Fluss et al. 2014). Back-splicing is favoured over canonical splicing when a downstream splice donor is brought into close proximity with an upstream splice acceptor. This can occur through ‘intron pairing’, where base pairing between complementary sequences within flanking introns (often inverted Alu repeats) induces looping of the RNA (Ashwal-Fluss et al. 2014, Jeck et al. 2013, Kramer et al. 2015, Liang and Wilusz 2014, Zhang et al. 2014). Alternatively, RNA binding proteins (RBPs) can promote back-splicing by binding to flanking introns and dimerizing (Ashwal-Fluss et al. 2014, Conn et al. 2015, Errichelli et al. 2017, Fei et al. 2017, Khan et al. 2016) or through stabilization of existing double-stranded RNA secondary structures formed through intron pairing (Li et al. 2017, Shen et al. 2022) (Fig. 4). These mechanisms are facilitated by faster rates of transcription, reducing the time taken for complimentary intron sequences and RBP responsive elements to become accessible (Shen et al. 2022, Zhang et al. 2016). The composition of resulting circRNAs varies, with some circRNAs containing exclusively exons (ecircRNA) and others containing exons and retained introns (exon-intron circRNAs) (Kristensen et al. 2019). This, in turn, dictates their subcellular localization and provides the basis for the diverse regulatory functions of circRNAs.

## Functions of circRNAs

### miRNA sponging

**Figure 5:**
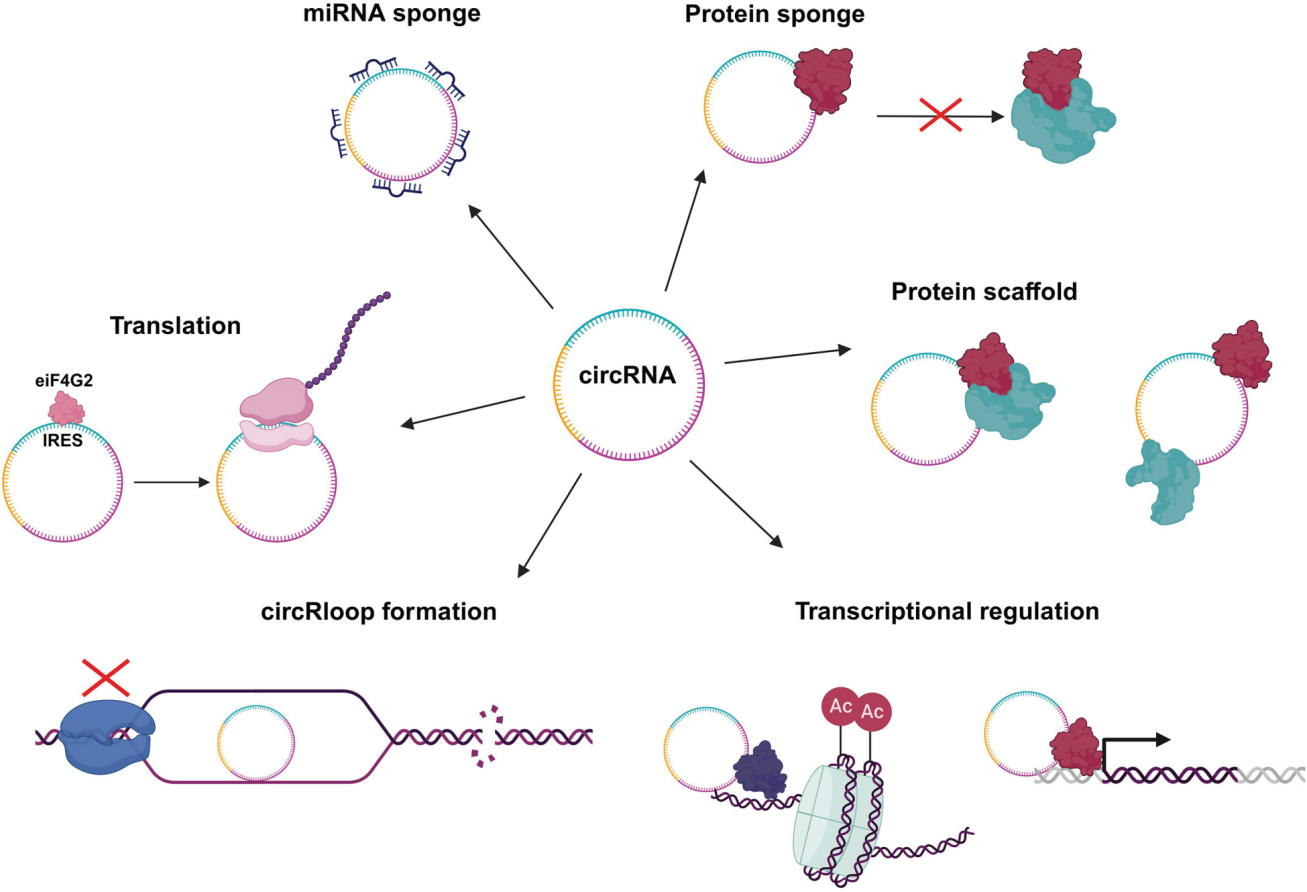
Diverse regulatory functions of circRNAs. circRNAs regulate cellular processes at multiple levels. circRNAs can regulate gene expression through the sponging and inhibition of miRNAs and through the recruitment of transcription factors and histone-modifying enzymes directly to target genes. circRNAs can also regulate protein function by acting as a protein decoy/protein sponge and can facilitate protein-protein interactions through protein scaffold formation. ORF-containing circRNAs that are m^6^A methylated or possess IRES sequences have the potential to be translated, producing circRNA-derived micro-peptides. Finally, circRNAs can also contribute to the formation of circR-loops and, consequently, genomic instability. Created in BioRender. Whitehouse, A. (2026) https://BioRender.com/m48c488.

Perhaps the most widely established function of circRNAs is their ability to sponge and inhibit miRNAs through a process known as the ‘Competing endogenous RNA (CeRNA) hypothesis’ (Jarlstad Olesen and S Kristensen 2021, Salmena et al. 2011) (Fig. 5). Here, cytoplasmic circRNAs utilize their own miRNA response elements (MREs) to competitively bind and sequester miRNAs, inhibiting their interaction with, and degradation of, target mRNAs (Salmena et al. 2011). This leads to the subsequent upregulation of target mRNAs. ciRS-7 contains 73–74 MREs specific to the miRNA, miR-7, and was the first circRNA reported to possess miRNA sponging capacity (Hansen et al. 2013, Memczak et al. 2013). Studies in zebrafish found that the overexpression of ciRS-7 produced the same phenotypic response as miR-7 knockdowns, highlighting ciRS-7 as a likely negative regulator of miR-7 (Memczak et al. 2013). The sponging of miR-873–3p by circTP63 in lung squamous cell carcinoma upregulates FOXM1 mRNA. This leads to the upregulation of downstream cell cycle regulators CENPA and CENPB and contributes to tumourigenesis (Cheng et al. 2019).

### Protein interactions

circRNAs also act as protein sponges, protein decoys, and protein scaffolds (Zhou et al. 2020) (Fig. 5). Protein sponging refers to the competitive binding and sequestration of proteins by circRNAs, often resulting in inhibition of protein function. circSMARCA5 sponges splicing factor SRSF1, inhibiting its ability to induce the alternative splicing of VEGFA pre-mRNA (Barbagallo et al. 2019). Alternatively, circNOTCH1 enhances the stability of its linear counterpart by acting as a protein ‘decoy’. circNOTCH1 competitively binds to the methyltransferase METTL14, inhibiting its interaction with NOTCH1 mRNA (Y. Shen et al. 2021). By simultaneously binding to multiple proteins, circRNAs also form scaffolds to facilitate protein interactions. circFOXO3 simultaneously binds to the cell cycle activator, CDK2, and tumour suppressor, p21, facilitating p21-mediated inhibition of CDK2 and subsequent cell cycle arrest (Du et al. 2016). Alternatively, circRNA scaffold formation has the potential to induce dissociation of natural protein complexes (Fang et al. 2019). circCcnb1 inhibits the formation of the CyclinB1/CDK1 complex through simultaneous binding to both proteins at positions that reduce their spatial proximity (Fang et al. 2019).

### Transcription regulation

Nuclear circRNAs typically function as regulators of transcription (Fig. 5). circPAIP2 and circEIF2J actively bind to RNA Polymerase II and U1 small nuclear RNA and direct them to the promoters of their parental genes (Li et al. 2015,Yang et al. 2017). circRNAs also mediate epigenetic regulation of gene expression through the recruitment of histone-modifying enzymes. For example, circMRPS35 mediates the recruitment of lysine-acetyltransferase, KAT7 to the promoters of tumour suppressors FOXO1 and FOXO3a, activating their transcription (Jie et al. 2020). More recently, circRNAs have been implicated in the formation of circR-loops via base pairing with their parental DNA (Conn et al. 2017, 2023, Holdt et al. 2018, Xu et al. 2020). CircR-loops induce halting of RNA Pol II leading to transcriptional inhibition (Conn et al. 2017, 2023, Xu et al. 2020) and are becoming increasingly recognized as mediators for genomic instability (Conn et al. 2023).

### Protein-coding capacity

A far less characterized function of circRNAs is their protein-coding potential (Fig. 5) (Lei et al. 2020). These circRNAs have the capacity to recruit the 43S ribosomal complex through internal ribosomal entry sites (IRES) (Marques et al. 2022) and the presence of *N*^6^-methyladenosine (m^6^A) modifications (Meyer et al. 2015, Yang et al. 2017), enabling their cap-independent translation. The first protein coding circRNA to be reported, circZNF609, possesses an IRES sequence, an AUG start codon derived from its parental gene, and a circularization-induced novel STOP codon (Legnini et al. 2017). Whilst some reports of circRNA-derived peptides have emerged (Liang et al. 2019, Pan et al. 2020, Zhang et al. 2018, Zheng et al. 2019), their exact role is yet to be fully elucidated.

## circRNAs and viruses

The ability of circRNAs to regulate cellular processes at a transcriptional, post-transcriptional, and post-translational level makes them valuable tools for viruses in the manipulation of host cells. It is not surprising, therefore, that several viruses have evolved to encode their own viral circRNAs (Ge et al. 2021, Sekiba et al. 2018, Tan et al. 2021, Toptan et al. 2018, Ungerleider et al. 2019) (Table 4). Whilst the exact mechanism of viral circRNA biogenesis remains unclear, recent evidence in herpesviruses suggests that, unlike cellular circRNAs, viral circRNAs are formed in a spliceosome-independent manner. Moreover, viral circRNA biogenesis occurs at non-canonical splice sites and is driven by alternative flanking motifs rather than the typical ALU repeats observed at cellular circRNA loci (Dremel et al. 2025). Viral circRNAs are seemingly formed via interactions with alternative trans-acting factors, including host RNA ligases (Dremel et al. 2025).

**Table 4:** List of virus-associated circRNAs, their origins, and their effects.

Virus	circRNA	Mechanism/function	Reference
EBV	circBART2.2 (V)	Activation of RIG1 leading to upregulation of PD-L1 and immune evasion.	PMID: 30 150 410 PMID: 34 321 242
	circLMP2 (V)	Sponging miR‐3908 regulating miR-3908/TRIM59/p53 axis. Induces stemness in EBV-associated gastric cancer.	PMID: 30 150 410 PMID: 34 257 379 PMID: 32 790 025
	circEBNA (V, up)	Unknown.	PMID: 30 567 979
	circRPMS1 (V)	Sponging of miR-203, miR-31, and miR-451. Promote proliferation and EMT in nasopharyngeal cancer cells.	PMID: 30 567 979 PMID: 31 695 488
KSHV	circv_IRF4 (V)	Unknown, possibly early stages of infection.	PMID: 30 150 410 PMID: 30 567 979 PMID: 31 911 496
	circPAN (V)	Unknown, possibly early stages of infection.	PMID: 30 150 410 PMID: 31 911 496
	circHIPK3 (C, up)	Sponging of miR-30c to induce upregulation of target DLL4. Cell cycle arrest and increased viral replication.	PMID: 35 239 998
	circARFGEF1 (C, up)	Sponging of miR-125a-3p to upregulate GLRx3. Promote lytic replication, cellular proliferation, and angiogenesis.	PMID: 33 539 420
	circRELL1 (C, up)	Antiviral, downregulates LANA and RTA viral proteins, and impairs virion production.	PMID: 30 455 306 PMID: 36 724 259
HPV	circE7 (V)	Activation of ACC1, impaired T cell activation (immune evasion). Translated to E7 oncoprotein	PMID: 39 366 979 PMID: 31 127 091
HBV	HBV-circ-1 (pgRNA derived) (V)	Interaction with CDK1 to enhance cell proliferation and viral replication.	PMID: 34 589 285 PMID: 29 765 512
	circATP5H (C, up)	Sponging miR138-5p to upregulate TNFAIP3 to promote viral DNA replication.	PMID: 33 173 336
	circRNF13 (C, up)	Sponging miR454-5p to upregulate TGIF2 to promote viral DNA replication.	PMID: 33 714 261

***Key: (C* *=* *cellular, V* *=* *viral, up* *=* *upregulated, down* *=* *downregulated)***

The repertoire of circRNAs implicated in viral infection is not limited to those encoded within the viral genome, as previous studies have highlighted the role of cellular circRNAs in host antiviral defence. Cellular circRNAs readily bind the immune factors NF90/NF110 in the cytoplasm under physiological conditions. Viral infection triggers a dissociation of the circRNA-NF90/NF110 complex, freeing NF90/NF110 to target and inhibit viral mRNAs (X. Li et al. 2017). In contrast, increasing studies corroborate novel pro-viral roles of circRNAs, and demonstrate that viruses can dysregulate host circRNAs to aid in their replication. These putative virus-host interactions are reported across several virus species, including KSHV (Harper et al. 2022, Tagawa et al. 2018, 2023, Yao et al. 2021), HSV-1 (Shi et al. 2018), SV40 (Shi et al. 2017), HPV (Zheng et al. 2018), HIV (Zhang et al. 2018), HBV (Jiang et al. 2020, Yu et al. 2020), and DENV (He et al. 2019); however, very few of these circRNAs have been fully characterized (Table 4).

## EBV

The EBV BART locus produces several, highly abundant circRNA isoforms (Ge et al. 2021, Toptan et al. 2018). Whilst the role of many of these circRNAs is yet to be elucidated, circBART2.2 has been implicated in promoting immune evasion. Mechanistically, circBART2.2 binds and activates RIG1, leading to the activation of downstream transcription factors IRF3 and NF-kB and the subsequent upregulation of the immune checkpoint protein, PD-L1 (Ge et al. 2021). EBV also encodes circRNAs derived from its LMP2 (Gong et al. 2020, Tan et al. 2021, Toptan et al. 2018), EBNA (Ungerleider et al. 2018, Ungerleider et al. 2019), and RPMS1 (Liu et al. 2019, Ungerleider et al. 2018, Ungerleider et al. 2019) gene loci, with some being implicated in the progression of EBV-associated cancers. Through their miRNA sponging capacity, circLMP2A and circRPMS1 upregulate the expression of stemness and EMT markers, respectively, enhancing the proliferative and migratory capability of EBV-infected cells (Gong et al. 2020, Liu et al. 2019).

## KSHV

In KSHV, circular transcripts originating from the vIRF4 locus and over 100 sense and antisense isoforms from the PAN locus have been identified (Toptan et al. 2018). Additionally, several lowly expressed circRNAs have been discovered from loci including ORF36, K4, ORF49, ORF69, K12, ORF71, and ORF72 (Tagawa et al. 2018, Toptan et al. 2018). circ-vIRF4 is expressed primarily during latency and is believed to exist as two isoforms, distinguished by the presence of an intron (Ungerleider et al. 2019). As a result, circ-vIRF4 displays both nuclear and cytoplasmic localization (Toptan et al. 2018). Despite these indications of possible function, the exact role of circ-vIRF4 and circ-PAN in KSHV infection remain largely unknown. Recent evidence, however, has shown that both viral circRNAs are readily packaged into new virions suggesting a possible role in the early stages of infection (Abere et al. 2020).

KSHV also induces the differential expression of over 280 host circRNAs during its lytic phase (Tagawa et al. 2018). One such circRNA, circHIPK3, is upregulated ∼3-fold upon lytic reactivation and is essential for viral DNA replication. circHIPK3 sponging of miR-30c induces the upregulation of Notch ligand, DLL4, and the subsequent downregulation of cyclins E1 and B1. Consequently, this promotes G1 arrest, a mechanism utilized by KSHV to reduce competition for cellular DNA replication machinery and enhance replication of its own DNA genome (Harper et al. 2022). Moreover, the upregulation of the circARFGEF1/miR-125a-3p/GLRx3 network is also induced upon KSHV lytic infection and is associated with increased proliferation and angiogenesis in infected endothelial cells (Yao et al. 2021).

Recent evidence suggests that KSHV is not solely responsible for host circRNA dysregulation upon infection and that some circRNAs may be upregulated as a mechanism of host defence. circRELL1 is significantly upregulated during KSHV lytic infection but appears to induce downregulation of KSHV markers RTA (lytic switch protein) and LANA (regulator of latency) and subsequently impairs infectious virion production, suggestive of antiviral activity (Tagawa et al. 2018, 2023).

## HPV

In HPV, expression of circE7 derived from the E6/E7 locus is associated with a novel mechanism of immune evasion. circE7 induces the epigenetic downregulation of galectin-9 through the activation of acetyl-CoA carboxylase 1 (ACC1). Reduced levels of galectin-9 subsequently impair CD8+ T-cell cytokine production and promote T-cell apoptosis (Ge et al. 2024). Additionally, it is hypothesized that circE7 can also be translated into the HPV oncoprotein E7. Findings by Zhao et al. (2019) corroborated this, as they showed how depletion of circE7 transcripts correlated with a >2-fold reduction in E7 oncoprotein levels and consequently impaired cell cycle progression (Zhao et al. 2019).

The functional role of circRNA-derived peptides, however, remains largely debated. Conflicting evidence surrounding the low levels of circE7 relative to that of the linear isoform E6*I has prompted questions regarding the functional relevance of the circE7-derived E7 protein. It is also debated whether the phenotype observed upon circE7 depletion is instead the result of co-depletion of E6*I (Wang et al. 2022, Yu and Zheng 2022). Nonetheless, this work provides evidence for a novel mechanism of HPV-mediated cell transformation.

## HBV

circRNAs derived from HBV pgRNA promote viral replication through interactions with cell cycle regulator, CDK1 (Sekiba et al. 2018, Zhu et al. 2021). Consequently, these circRNAs are implicated in the enhanced proliferation of HBV-associated hepatocellular carcinoma cells (Zhu et al. 2021). HBV also induces the dysregulation of host circRNAs and their associated ceRNA networks. Dysregulation of the circATP5H/miR138-5p/TNFAIP3 and circRNF13/miR454-5p/TGIF2 networks by HBV is essential for HBV viral DNA replication and the production of hepatitis B surface antigen (HBsAg) and hepatitis B e antigen (HBeAg), which contribute to HBV virion entry and immune evasion (Chen et al. 2021, Jiang et al. 2020).

## HSV and SV40

Virus-induced host circRNA dysregulation during HSV-1 and SV40 infections has been implicated in the dysregulation of p53 and Wnt cellular signalling pathways, thereby enhancing cell cycle progression and survival (Shi et al. 2017, 2018). Additionally, these dysregulated circRNAs may also contribute to immune evasion, with components of the JAK-STAT pathway highlighted as predicted targets (Shi et al. 2017, 2018).

## tRFs

Transfer RNAs are subject to degradation, and for a long time their cleavage products were disregarded, deemed only as by-products of non-specific fragmentation. However, there is a growing body of literature uncovering regulatory roles of tRNA-derived RNA fragments (tRFs) in gene expression both at transcriptional and post-transcriptional levels and directly implicating tRFs in various biological and pathophysiological processes (Krishna et al. 2021, Kumar et al. 2016a, Yu et al. 2020).

## Classification and biogenesis of tRFs

**Figure 6:**
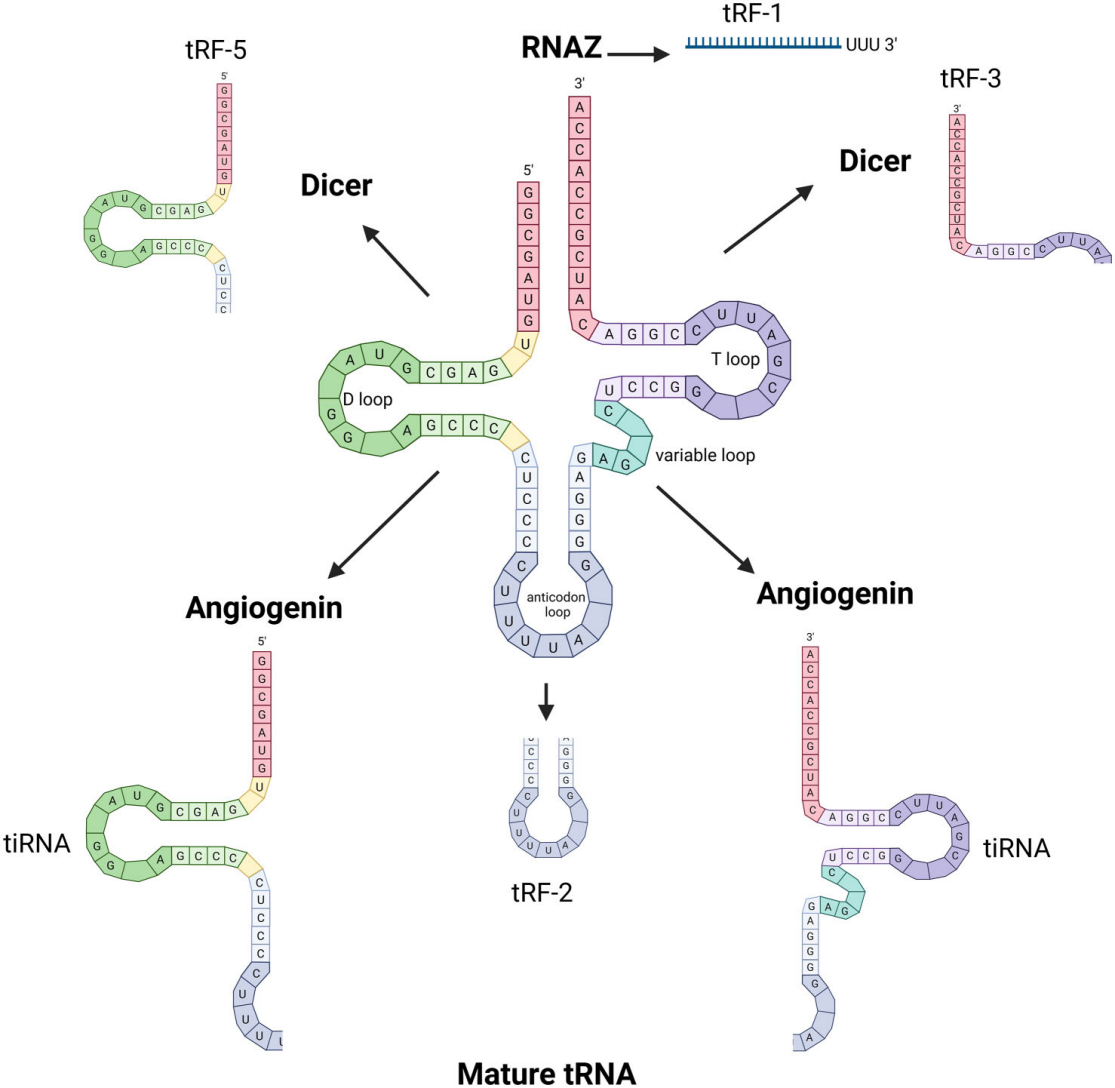
Classification and biogenesis of tRFs. Pre-tRNA undergoes enzymatic processing to generate mature tRNA. tRF-1 is generated by RNase Z-mediated cleavage of pre-tRNA. tRF-5 s and tRF-3 s are generated from the digestion of mature tRNA by Dicer. Angiogenin cleavage in the anticodon loop of mature tRNA generates tiRNAs. Created in BioRender. Whitehouse, A. (2026) https://BioRender.com/7fjfvnb.

tRFs are primarily categorized based on where they map along the primary or mature tRNAs from which they are derived (Fig. 6). The trimming of trailer sequences during tRNA maturation generates the first class of tRFs, termed tRF-1s. These are approximately 18–20 nucleotides in length and contain the characteristic poly-UUU tail at the 3’ end of primary transcripts (Katsaraki et al. 2019, Kumar et al. 2016a).

tRNA halves or tiRNA, are generated by a single nick in the anticodon loop of mature tRNAs, creating RNA fragments that are 31–40 bases long. It is speculated that the enzyme responsible for this site-specific cleavage is Angiogenin, and the resulting tRFs are categorized either as 5-tiRNA or 3-tiRNA depending on which end of the mature tRNA sequence they encompass (Xie et al. 2020, Yamasaki et al. 2009).

Mapping along the 5’ end of mature tRNAs are tRF-5 s. These are generally 14–30 nucleotides in length and are generated from cleavage in the D loop/anticodon stem (Kumar et al. 2016a). Similarly, tRF-3 s are the products of cleavage in the T loop. There are discrepancies in literature about the biogenesis of tRF-5s and tRF-3s. Several studies have shown that Dicer is responsible for the processing of these tRFs (Cole et al. 2009, Kumar et al. 2016b, Yu et al. 2020). Conversely, following a global sequencing analysis of small RNA in wild-type and Dicer knockout cells, Langenberger et al. (2013) observed no decrease in the abundance of tRFs in human, mouse, *Drosophila*, or *Schizosaccharomyces pombe* cells (Langenberger et al. 2013). Therefore, it appears there could also be other mechanisms driving the generation of tRF-5s and tRF-3 that are yet to be uncovered.

Other more obscure classes of tRFs include tRF-2s and i-tRFs. tRF-2s encompass the anticodon loop, and i-tRFs are derived from the internal body of mature tRNAs but exclude the 5’ and 3’ terminals. The mechanisms involved in the processing of both these classes remains unknown (Kumar et al. 2016a).

## Functions of tRFs

Due to their comparable sizes, tRFs were long mistaken for other ncRNA species, such as miRNAs and piwi-interacting RNAs (piRNAs). This structural similarity was proposed to equate to a functional overlap between these small RNAs. An early study conducted by Maute et al. (2013) was the first to validate this hypothesis, reporting that a tRF-3 derived from tRNA-Gly^CCC^ functioned in a miRNA-like manner (Maute et al. 2013). Similar to miRNAs, these tRFs were generated in a Dicer-dependent mechanism, physically associated with Argonaute (Ago) proteins, and employed a sequence complementarity mechanism to inhibit translation. Differential expression analyses of tRF levels in breast cancer cell lines have since revealed that tRNA-Glu, tRNA-Asp, and tRNA-Gly-derived tiRNAs utilize post-transcriptional repression to suppress tumour progression (Goodarzi et al. 2015). Only recently are we coming to appreciate the global impact of tRF-mediated gene regulation and the crucial implications for complex cellular and pathophysiological processes.

## tRFs and viruses

### HTLV1

Viruses have adopted ingenious methods to incorporate tRFs in immuno-evasion (Table 5). In some cases, viruses also manipulate tRFs to enhance their replication (Nunes et al. 2020). A prime example is human T-cell leukaemia virus type 1 (HTLV1) infection, where another facet of the functional diversity of tRFs is observed. A proline-derived tRF-3, termed tRF-3019, was identified with sequence complementarity to the HTLV1 promoter, and functional analyses revealed that the tRF-3 had sufficient priming activity to facilitate viral genomic replication (Ruggero et al. 2014). This novel role of tRFs highlight their potential to act as a novel antiviral target.

**Table 5:** tRFs implicated in viral biology.

Virus	tRF	Mechanism/function	Reference
HTLV1	tRF-3019 (C, up)	Has priming activity for the HTLV1 reverse transcriptase and promotes HTLV-1 replication.	PMID: 24 403 582
RSV	tRF 5-Glu^CTC^ (C, up)	Functions in a miRNA-like manner to silence gene expression and facilitate viral replication.	PMID: 26 156 244
HCV	tRF 1-Ser^TGA^ (C, up)	Sequesters the chaperone protein La/SSB and prevents it from stabilizing viral transcripts, thereby inhibiting viral replication.	PMID: 31 504 775
DENV	tRF-3 s (C, up)	Dysregulates the PI3K-AKt signalling pathway to promote DENV replication.	PMID: 39 112 524
MHV68	tRF-3 s (V, up)	Yet to be elucidated.	PMID: 38 746 336

***Key: (C* *=* *cellular, V* *=* *viral, up* *=* *upregulated, down* *=* *downregulated)***

### RSV

RSV uses the gene silencing function of tRF-5s from tRNA-Glu^CTC^ to facilitate viral replication (Wang et al. 2013). In small alveolar epithelial cells infected with RSV, the tRNA-Glu^CTC^-derived tRF-5 was shown to possess miRNA-like *trans-*silencing capacity, with target gene inhibition promoting viral replication. Interestingly, the same tRFs are also upregulated upon cytomegalovirus infection of fibroblasts (Soares et al. 2015). RSV exemplifies the inventive ways viruses have evolved to manipulate host biology in favour of their own replication, as Choi et al. (2023) have recently shown using novel mass spectrometric-based sequencing methods that RSV infection modifies the methylation profile of parental tRNAs to promote their cleavage and subsequent tRF biogenesis (Choi et al. 2023).

### HCV

Some tRFs also exhibit antiviral roles during infection. For instance, a tRF-1 derived from pre-tRNA-Ser^TGA^ shows inhibitory effects on HCV pathogenesis. This tRF sequesters the chaperone protein La/SSB, which would otherwise stabilize nascent HCV transcripts, facilitating ribosomal entry. As a consequence, tRF activity destabilizes viral transcripts and inhibits HCV gene expression (Cho et al. 2019).

### DENV

Comparative analyses of RNA sequencing data sets in DENV-infected Huh7 cells highlighted an increase in tRF-3 levels following DENV infection, with KEGG functional analysis revealing a significant enrichment in the PI3K-AKt signalling pathway. This suggests that tRF-3s could be integral to the dysregulation of signalling pathways during DENV infection, as the PI3K-AKt network is known to be heavily dysregulated during the course of DENV infection (Lahon et al. 2021).

### MHV68

By coupling ordered two-template relay sequencing technology with tRNA-specific bioinformatic analytics, Manning et al. (2025) successfully developed a method for simultaneously quantifying full-length tRNAs and tRF levels in a single sequencing profile. This combinatorial approach was used to investigate differential tRNA and tRF expression levels in murine herpesvirus 68 (MHV68)-infected fibroblasts. Interestingly, they identified 3’ fragments from seven viral tRNA (virtRNA) molecules, representing the first identified case of viral-derived tRFs (virtRFS). However, whether or not these virtRFs are functional remains to be elucidated (Manning et al. 2025).

Moreover, their differential analyses demonstrated that pre-tRNAs do not exclusively generate tRF-1s, as literature currently suggests but serve as precursors for the generation of diverse tRF subgroups. The authors postulated that this unique tRF signature was a characteristic of gammaherpesvirus infection, further highlighting the impact of viral infection on host tRNA expression and processing.

## Conclusions and future perspectives

Evidently, ncRNAs play important and diverse roles in the development and progression of various diseases, and their complex contribution to viral infection is becoming increasingly apparent. Despite improvements in our ability to detect dysregulated ncRNAs, few have been experimentally validated and functionally characterized in the context of viral infection, particularly tRFs and piRNAs, for which research remains largely in its infancy. The full extent of the myriad of ncRNAs manipulated by viruses is yet to be uncovered. Nonetheless, ncRNAs have cemented their relevance in virology, presenting a novel opportunity for the design and development of diagnostic tools and effective therapeutic interventions.

### ncRNAs as biomarkers

ncRNAs are typically expressed in a cell-type-specific manner and their dysregulation is a defining feature in many diseases. As such, ncRNAs are gaining increasing traction as promising candidates for the development of prognostic biomarkers (Eldakhakhny et al. 2024). This is further supported by the discovery that ncRNAs are highly enriched within exosomes. As exosomes are readily excreted by cells into bodily fluids, this offers a distinct opportunity for minimally invasive prognostic methods using liquid biopsies (Eldakhakhny et al. 2024). Several studies have already identified a correlation between dysregulated ncRNA expression and disease progression/patient prognosis (Goh et al. 2022, Parashar et al. 2024, Spanos et al. 2023, Weng et al. 2022, Zhao et al. 2024). In cervical cancer, higher expression of the lncRNA ANRIL correlated with enhanced risk of lymph node metastasis (Zhang et al. 2017). Whereas low expression of the tumour suppressor circRNA, circPVT1 was identified as a common characteristic of T4-stage gastric tumours and was associated with shorter overall survival (Chen et al. 2017).

ncRNAs also offer an opportunity to monitor drug resistance. High expression of circCRIM1 in nasopharyngeal carcinoma correlates with resistance to docetaxel chemotherapy. Patients with low circCRIM1 expression were found to have increased sensitivity to docetaxel treatment and reduced risk of recurrence, whilst patients with high expression showed no response (X. Hong et al. 2020). Similarly, upregulation of lncRNA HOTAIR promotes resistance to cisplatin treatment in gastric cancer by promoting cell survival through enhancing PI3K/Akt and Wnt/β-catenin signalling pathways (Cheng et al. 2018).

Research into biomarkers to monitor viral infections and associated diseases has largely focused on the use of dysregulated host ncRNAs. Early evidence has identified 5' tRNA halves (5-tiRNA) as potential biomarkers of HBV-associated tumourigenesis, as glycine-derived 5’tiRNAs were consistently downregulated in HBV-infected HCC patients (Selitsky et al. 2015). The differential expression of multiple host ncRNAs during SARS-CoV-2 infection also offers an opportunity to monitor disease severity (Ranches et al. 2025). In contrast, the potential of virus-encoded ncRNAs has been less extensively explored, primarily because viral genomes encode far fewer ncRNAs by comparison. Limitations therefore arise in the development of biomarker panels that are often required for accurate disease monitoring. Additionally, host responses to viral infection are typically more relevant to disease prognosis and these pathways are often more established. Nevertheless, virus-encoded ncRNAs harbour much promise. For instance, viral ncRNA have the potential to overcome current obstacles associated with diagnosing herpesvirus infection, as latency-associated suppression of viral mRNAs has long complicated viral detection using currently available diagnostics. Indeed, multiple studies have highlighted the potential of latently expressed, EBV-encoded miRNAs as biomarkers of chronic infection (Hartung et al. 2019, Kawano et al. 2013, Movassagh et al. 2019). Despite mounting evidence supporting the potential of ncRNAs as disease biomarkers, they are yet to be integrated into the clinic.

### ncRNAs as therapeutic targets

Due to their diverse regulatory functions, ncRNAs are also emerging as novel therapeutic targets. Methods of targeting ncRNAs include the use of antisense oligonucleotides (ASOs). ASOs are small single-stranded RNA molecules capable of binding to target ncRNAs and inhibiting their function (Chen et al. 2022). Miravirsen is an anti-miRNA ASO which has demonstrated success in phase II clinical trials as an antiviral therapy for HCV (Ottosen et al. 2015). Mechanistically, Miravirsen specifically targets miR-122 inhibiting its interaction with HCV genomic RNA (Ottosen et al. 2015). However, such nucleic acid-based therapeutics are not without their limitations, including high immunogenicity (due to activation of toll-like receptors) (Nappi 2024). Therefore, alternatives such as small molecule inhibitors have also been explored (Monroig-Bosque et al. 2018).

### ncRNAs as therapeutic agents

In addition to targeting ncRNAs, mounting evidence supports the use of ncRNAs as therapeutics themselves. At present, research has largely focused on the delivery of therapeutic miRNAs, as their small size and efficient, specific regulatory potential makes them prime candidates in drug discovery ventures. However, the successful delivery of miRNAs to cells faces several challenges, top of which is their susceptibility to degradation (YiRan Li et al. 2025). The encapsulation of miRNAs within lipid nanoparticles has presented as a possible solution. One such therapy is MRX34, a lipid nanoparticle cancer therapy containing mimics of miR-34a. Whilst MRX34 was shown to successfully deliver miR34a mimics to target tumours with potent functional activity in a Phase I trial, significant concerns arose surrounding immunogenic toxicity (Hong et al. 2020).

More recently, attention has turned to the possibility of harnessing the natural function of extracellular vesicles as carriers of ncRNAs. As such, miRNA-enriched EVs are being explored for their treatment potential (Menjivar et al. 2024). Some success has been observed in this regard, as the delivery of miR-424 in MSC-derived EVs has been shown to successfully inhibit cell proliferation and migration in ovarian cancer cells (Li et al. 2021). Additionally, in a glioblastoma mouse model, treatment with MSC-derived EVs carrying miR-124a led to complete tumour regression in 50% of mice (Lang et al. 2018).

Future efforts are devoted to the development of engineered EVs enabling the delivery of specific miRNA cargoes and should also explore means of delivering other ncRNA species (Menjivar et al. 2024). The utilization of ncRNAs as therapeutics is a promising field; however, some major challenges must still be overcome, including off-target effects and high immunogenicity as well as a need to optimize efficient delivery (Seyhan 2024). Nonetheless, the possibility of delivering antiviral ncRNAs to infected cells is an exciting concept.

In summary, the dynamic expression of host and viral ncRNA species during viral infection is an intriguing discovery but the full extent of these regulatory networks remains elusive. Functional characterization of these dysregulated ncRNA species could provide a solid framework for the discovery of novel therapeutic targets, as well as potential biomarkers of virus-associated tumourigenesis.
